# Making sense of street chaos: an ethnographic exploration of homeless people’s health service utilization

**DOI:** 10.1186/s12939-019-1002-6

**Published:** 2019-07-23

**Authors:** Austin O’Carroll, David Wainwright

**Affiliations:** 1 North Dublin City GP Training Scheme, Dublin 7, Ireland; 2 University of Bath, Dublin 7, Ireland

## Abstract

**Background:**

Homeless people have poor health and mortality indices. Despite this they make poor usage of health services. This study sought to understand why they use health services differently from the domiciled population.

**Methods:**

Ethnographic observations were conducted at several homeless services, in Dublin. This was supplemented with 47 semi-structured interviews with homeless people and two focus groups of homeless people and hospital doctors. A critical-realist approach was adopted for interpretation of the data.

**Results:**

Homeless people tended to present late in their illness; default early from treatment; have low usage of primary-care, preventative and outpatient services; have high usage of Emergency and Inpatient services; and poor compliance with medication. They tended to avoid psychiatric services. A number of external barriers were identified. These were classified as physical (distance) administrative (application process for medical care; appointments; queues; the management of addiction in hospital; rules of service; and information providing processes); and attitudinal (stigma; differing attitudes as to appropriate use of services. A new form of barrier, Conversations of Exclusion was identified and described. Internalised barriers were identified which were in nature, either cognitive (fatalistic, denial, deferral to future, presumption of poor treatment or discrimination, self blame and survival cognitions) or emotional (fear; embarrassment, hopelessness and poor self-esteem). Generative mechanisms for these factors were identified which either affected participants prior to homelessness (marginalization causing hopelessness, familial dysfunction, substance misuse, fear of authority, illiteracy; mental health; and poor English) or after becoming homeless (homelessness; ubiquity of premature death; substance misuse; prioritization of survival over health; threat of violence; chaotic nature of homelessness; negative experiences of authority; and stigma.

**Conclusions:**

An explanatory critical realist model integrating the identified generative mechanisms, external and internalised barriers was developed to explain why the Health service Utilization of homeless people differs from the domiciled populations. This new model has implications for health service policy makers and providers in how they design and deliver accessible health services to homeless people.

## Background

Internationally it has been found that homeless people have significantly higher mortality rates than the general adult population [1, 2]. In comparing the slope in increasing morbidity associated with health inequalities, the health experience of homeless people has been described as ‘more akin to a cliff’ [3]. Homeless people face a disproportionate burden of infectious diseases including HIV, Hepatitis and Tuberculosis and chronic diseases including higher rates of asthma, heart disease, stroke, epilepsy and multi-morbidity [3–5]. Homeless people also have high rates of mental-ill health with high rates of schizophrenia, depression and anxiety. This increased mental illness burden has resulted in higher suicide rates [6]. Homeless people also have much higher rates of alcohol and drug-addiction than the general population [5, 7].

Irish studies have found similar high rates of addiction, poor physical and mental health. O Reilly et al. (2013) found alcohol and drug misuse as pervasive with 41 and 59% of homeless people reporting addiction to alcohol or drugs respectively and 24% reporting intravenous drug misuse. Eighty-one percent had a diagnosis of at least one chronic physical health condition, 58 % at least one mental health diagnosis and 27 % a blood-borne-viral infection. Twenty-eight percent had suicidal thoughts in the previous 6 months and 36 % had attempted suicide at some stage in their lives [8].

It is well recognised that the decision to attend a health service cannot be predicted from the symptoms a person is experiencing. Only between 1 in 18 to 1 in 37 of symptoms result in a consultation with a health provider. This has been termed the clinical iceberg. It has further been found that there is little correlation between the potential seriousness of a symptom and the likelihood of whether the person decides to consult or not. A wide array of factors have been identified that influence the evaluation of whether symptoms require medical advice or not [9].

The decision to present with a symptom or has been variously described in the literature under an array of conceptual frameworks including consultation-triggers; consultation-behaviours; health service utilization; health-seeking-behaviour; and healthcare-seeking-behaviour. The first three of these approaches concentrate on endpoint usage of a health service whilst the other models look at a range of methods for managing symptoms including strategies outside the formal health system (e.g. self-management; attendance at alternative practitioner etc.). The chosen model for this research is Health-service-Usage (HSU) which refers to the process whereby individuals make a decision to attend a health service.

Despite their poor health profile, internationally, homeless people use health services differently to the housed population in a manner that results in delayed or no treatment for their many health problems [9]. They have a tendency to avoid health services and as a result endure a high burden of the untreated health conditions [11, 12]. They have very poor compliance rates in comparison to housed populations [11, 12]. These factors both contribute to a tendency to present late on in the course of a disease when their symptoms are critical and overwhelming [11, 12]. They also have a propensity to default from treatment including leaving hospital wards and ED before completion of treatment and missing outpatient appointments [11, 12]. This HSU pattern results in significantly raised costs for the health service [13]. This pattern of HSU has been replicated in Irish Research. Between 25 and 45% Irish homeless people do not have access to a general practitioner [8]. ED attendances are higher for homeless people than the general population (3.00 vs 0.16 attendances per year) as are hospital bed days (4.4 vs 0.3 per annum). Homeless people were also more likely to leave ED prior to assessment (40% vs 15% of ED attendances per annum) and also to leave hospital prior to completion of treatment (15% vs 2% admissions per annum) [14].

This seeming mis-match of health needs and seeming inappropriate and ineffective health service usage can seem chaotic and counter-productive to non-homeless people. There has been little research into understanding these seemingly inefficient HSU behaviors both in Ireland and internationally. Understanding why homeless people have such seemingly counterproductive HSU behaviours would allow us develop strategies to improve access to health services. Flato et al. (2010) pointed out that *“the life … of a homeless person appears chaotic from the standpoint of the domiciled citizen, yet the social and economic strategies of homeless people can be understood as the outcome of conscious deliberation and as rational in light of their difficult situation.”* [15] The aim of this research was to describe the HSU of homeless people in Dublin and to gain insight into why their HSU differs from the domiciled population.

## Methods

Critical-realist ethnography was the chosen methodology for this research. The rationale for choosing ethnography was that it offers the advantage of facilitating the observation of behaviour and contemporaneously exploring the rationale for the behavior and the contextual features that shape decisions and behaviours. This is unique to ethnography as quantitative methods primarily explore behaviours while qualitative methods (other than ethnography) primarily explore health beliefs and intentions [16, 17].

Bhaskar developed the critical-realist position which sits midway on a continuum from positivism to social constructivism. Critical realism understands the world as being stratified into domains of the real (i.e the ‘generative’ structures and mechanisms that generate events, relations and discourses) which is inaccessible to our clouded perception; the actual (i.e. the events, relations and discourses that are caused by these generative mechanisms); and the empirical i.e. our perceptions of the events, relations and discourses. Generative mechanisms are not understood as conjunctivist causes but rather as the social processes that produce social and behavioural tendencies [18, 19]. Thus the findings of critical realist research cannot predict individual homeless person’s behaviours but rather help understand why the population as a whole tends to have a particular HSU.

Critical-realists are methodological pluralists, accepting that a variety of methods may be required to gain a fuller understanding of a social phenomena and that will enable the creation of better explanatory theories at the generative level [18, 20]. Homelessness can either be interpreted broadly including a number of subcategories from insecure or inappropriate accommodation to rough sleeping or narrowly as ‘literal homelessness which refers to those rough sleeping or using hostels or temporary accommodation. This research focused on literal homelessness.

Data were gathered at three fixed sites in Dublin (a drop-in centre for homeless people; a food-hall for homeless people; a local ED; and an outreach service which worked with rough sleepers. A number of participants who were engaged with at the fixed sites or with the outreach team were further engaged with individually either on the street or at other homeless services. There were several reasons for choosing these sites. Firstly, they offered a wide typographical range of homeless people (i.e. based on gender; ethnicity; rough sleeping/accommodation status; and social problems that are often found in homelessness e.g. drug or alcohol addiction or mental illness). The drop-in centres were attended by a predominantly drug-using long-term homeless population who would have stayed in hostels. The food hall would be attended by a wider range of homeless individuals including those who use drugs, but also recently homeless people, street drinkers, economically homeless people and migrant homeless people. This site also had a drop-in primary care service that allowed the exploration of HSU at point of usage. Many rough sleepers do not attend either service and so it was decided to go out with the rough sleeper team to access this population. Lastly, attending at the ED allowed ethnographic research engagement with homeless people actually attending a secondary care service and offered a perspective on their use of these services. Multi-sited ethnography is a recognised form of ethnography that from a critical realist perspective improves validity as it is less likely to be biased by the vagaries of a single site [21]. Overall, 142 participants were recruited to the ethnographic study (47 for the semi-structured interview (31 male;16 female including 2 non-Irish participants); 69 participants I came across during ethnographic work (47 male;22 female including 7 non-Irish participants) and 26 for the focus groups (13 male;13 female). The gender and ethnicity profile is similar to other Irish studies of the homeless population [8].

This work took place for 5 h each week over 15 months. Intermittent shortened ethnographic time mode is a recognized ethnographic research approach [22]. The researcher visited each site and introduced themselves to staff and service users and explained the purpose of the research. Each day he would return and ‘hang out’ at the site and foster relationships with participants. At night with the rough sleeper team the researcher would form bonds with particular clients he ended up engaging with while accompanying the team. The researcher was known as a doctor by many staff and participants. To address this potential research bias the researcher adopted Wasserman & Clarks approach of making evident their position and background as a doctor. This honesty allowed the development of a relationship based on trust where commonalities between the researcher and participants could emerge while differences could fade into the background. Wasserman described this process in his research where he was known as the Professor while engaging with a group of homeless people who accepted him into their group. Throughout the process the researcher remained authentic to their personal views e.g. when discussion on homosexuality arose the researcher disagreed with the stigmatizing attitudes of some of the participants. This authenticity was not used to persuade participants to the researchers perspective but rather to improve trust that the participants could identify the researcher was being true and honest [23].

In addition,47 semi-structured interviews (31 male, 16 female) were conducted (ranging between 10 and 50 min) with homeless participants at the three sites as well as by visiting various hostels that had been identified when assigned to the Rough Sleeper team. Interviews were conducted at a site of choosing of the participant (e.g. a coffee shop or a drop-in centre). Sixteen appointments were unsuccessful, where the participant had either left the hostel, did not want to get out of bed or did not turn up. These were followed up and 10 of these participants were successfully recruited leaving six whom that were not interviewed The demographics and behavioural typologies of the interviewees is included in Tables 1 and 2.

**Table 1:** Categorization of Influences on Help-Seeking Behaviours for the General Population [8]

○ Socio-demographic factors: ● Age ● Gender ● Ethnicity ○ Socioeconomic factors: ● Social Class ● Unemployment ● Familial and social network influences ○ Psychological Factors: ● Perceived susceptibility. ● Perceived Severity ● Knowledge about illness and information seeking behaviour. ● Belief in the effectiveness of healthcare. ● Belief in the effectiveness of self-care ● Stressful Life Events. ● Perceived benefits and costs of obtaining healthcare. ○ The Organization of Healthcare: ● Distance from Surgery ● Appointments Systems. ● Doctor Initiated Consultations. ● Access to Emergency Departments.

**Table 2:** Demographics, substance misuse, mental health and blood borne Infectious status of semi- structured interview participants (not including ethnographic and focus group participants)

Participant ID	Sex	Age	Hx IV Drug Use	Hx of Benzo Abuse	Hx Alcohol Misuse	Hx of Methadone Treatment	Hx Mental Ill Health	Hx HIV	Hx Hep C	Hx of being in care	Ethnicity	Housing
1	M	33	Y	Y					Y		Irish	Hostel
2	M	35	Y	Y		Y	Y		Y		Irish	Hostel
4	F	33		Y							Irish	Hostel
5	M	48	Y			Y	Y	Y	Y		Irish	Hostel
6	M	63	Y			Y				Y	Irish	Hostel
10	M	30	Y	Y							Irish	Hostel
13	M	31	Y	Y		Y					Irish	Hostel
18	F	34	Y	Y	Y		Y		Y		Irish	Hostel
19	M	31	Y	Y		Y	Y				Irish	Hostel
21	M	23	Y	Y	Y	Y	Y			Y	Irish	Hostel
23	F	21	Y			Y					Irish	Hostel
26	F	31	Y	Y					Y		Irish	Hostel
27	M	23	Y	Y	Y	Y	Y			Y	Irish	Hostel
28	F	28	Y	Y			Y			Y	Irish	Hostel
30	F	45					Y				Irish	Hostel
33	M	39	Y	Y		Y	Y	Y	Y		Irish	Rough Sleeper
34	M	32	Y	Y		Y	Y	Y			Irish	Hostel
39	M	31	Y	Y		Y					Irish	Hostel
40	M	37	Y	Y	Y		Y			Y	Irish	Rough Sleeper
41	F	25	Y	Y			Y		Y		Irish	Hostel
42	M	49	Y	Y	Y	Y	Y		Y		Irish	Hostel
43	M	31	Y	Y					Y		Irish	Hostel
45	F	50	Y	Y					Y		Irish	Hostel
46	M	31	Y	Y			Y		Y		Irish	Hostel
50	M	30	Y	Y		Y	Y				Irish	Hostel
53	M	42	Y	Y					Y		Irish	Hostel
54	M	37	Y		Y	Y	Y				Irish	Rough Sleeper
60	M	38	Y		Y	Y	Y		Y		German	Rough Sleeper
61	F	40	Y	Y	Y	Y	Y				Irish	Hostel
62	M	36	Y	Y					Y		Irish	Hostel
63	F	24	Y	Y					Y		Irish	Hostel
64	F	44	Y	Y			Y		Y		Irish	Hostel
65	M	37	Y	Y		Y			Y		Irish	Hostel
66	F	35	Y	Y		Y	Y				Irish	Hostel
67	M										Irish	Hostel
68	F	30	Y	Y			Y		Y		Irish	Hostel
69	M	37					Y		Y		Irish	Hostel
70	M	45	Y	Y		Y					Irish	Hostel
71	M	26	Y	Y	Y						Irish	Hostel
72	M	39	Y	Y					Y		Irish	Hostel
73	F	20	Y	Y		Y					Irish	Hostel
74	M	40	Y								Portugal	Hostel
75	F		Y	Y	Y		Y	Y	Y		Irish	Hostel
76	M	23					Y				Irish	Hostel
77	M	59			Y		Y			Y	Irish	Rough Sleeper
78	F	48	Y	Y	N	Y		Y			Irish	Hostel
79	M	53	Y	Y	N	Y		Y			Eastern Europe	Hostel

Two focus groups were conducted with 14 homeless people and 12 hospital-based emergency doctors that were recruited through a GP training scheme. These doctors were interested in learning about why homeless people made such poor usage of health services and agreed to participate in a focus group with homeless drug using participants recruited from the Ana Liffey Project. The purpose of including this in the research was it offered an opportunity to explore the dynamic between hospital based doctors and homeless people in hospital settings and deduce how that affected HSU.

Hammersly & Atkinson’s (2007) suggestion that ethnography should have a funnel structure, where an initial broad area of research is narrowed down to specific areas of interest, was adopted. Field notes and memos were maintained during this period of focusing on the research question which helped identify research avenues worth exploring [24]. The analysis was both continuous and iterative as described by Glaser & Strauss [25]. A concomitant reflective process was conducted to identify and address any personal biases as well as biases introduced by the researcher’s presence in the field. All field notes, the focus groups and the initial 10 interviews were transcribed by the researcher so as to enable immersion in the data and subsequent interviews were transcribed professionally. The data were loaded onto NVIVO. Saldana’s approach to coding was utilised to move from initial codes to conceptual categories [26]. A code is a ‘researcher generated construct that symbolizes and thus attributes meaning to each individual datum for later purposes of pattern detection’ An inductive approach was applied whereby the analysis was driven by the data, i.e. the codes and themes were not preformed prior to data collection and/or analysis, but derived from immersion in the data itself. Initial open coding was re-coded using eclectic-coding. Attribute coding was assigned to both participants and ethnographic sites and a mixture of in-vivo, open, emotional, sub-coding and magnitude coding for the remaining data. This resulted in the production of 741 codes. A review of these codes allowed grouping into a group of themes using Glaser & Strauss’s constant comparative method, i.e. continuously comparing data appearing in categories, trying to identify differences between it and other data within that category and similarities with other categories [25]. This resulted in the development of sub-categories; merging categories; and the transformation of vague categories into more clearly defined ones. Concept formation occurs when the categories become abstracted from reality into conceptual categories. Categories were refined and relationships between categories were explored by referral to the data. In developing the final explanatory model the critical realist technique of retroduction was adopted. Retroduction was suggested by Roy Bhaskar as a methodological approach in critical realist research for identifying generative structures and mechanisms via observations at the empirical level. Retroduction involves creatively postulating as to possible generative structures or mechanisms that could generate the empirical observations of the researcher. The researcher then seeks to find which of those hypothetical structures or mechanisms produces a ‘best fit’ explanation for the range of empirical observations [18].

Throughout this process the researcher sought to ensure the trustworthiness of the findings. Lincoln & Guba (1985) identified four elements of trustworthiness i.e. credibility [how congruent are findings with reality]; transferability (how transferable are the findings to other sites or populations); dependability (would another researcher with the same data produce the same findings) and confirmability (how much influence has the researcher’s bias had on the findings) [27].

Shenton (2004) suggested a number of approaches to ensuring these four criteria are respected [28]. Credibility was maintained by using methodological triangulation of findings (between data generated from ethnographic observations, semi-structured interviews and focus groups). In addition, negative cases were reviewed and lastly, findings were compared to those identified in previous research studies. Transferability was improved by the use of multiple research sites. Dependability was improved through triangulation and by enumerating the number of sources for each category and sub-category. Lastly, confirmability was improved by recording reflections of the researchers personal views and their impact on the research site as suggested by Saldana [26].

## Results

The research firstly, found that the HSU of homeless people mirrored that described in the literature.

### Delayed presentation for treatment. (39 sources/75 references).

Many Participants delayed presenting for treatment. Participant-19: *‘It just.....I didn’t think I could die or if I cared...I kind of waited and waited ‘till the last minute before I’d do something about it.’*

There were 14 incidents where Participants delayed presenting with serious conditions e.g. Participant-43 had decided not to go to ED after a stranger had half bitten his ear off despite knowing of the risk of blood-borne-infections.

There also were 11 specific accounts by Participants recounting how they had delayed presenting with health problems. Participant-6: ‘*I had blood poisoning … and blood clots in my leg and I actually walked around for … a week and a half because I didn’t know what to do.’*

### Defaulting from treatment prior to completion. (33 Sources/78 references)

Many Participants reported defaulting prior to completion of treatment. Some were inpatients in hospital and left before their condition for which they were admitted resolved. Participant-12 had pneumonia and did not know where to go so she let it get very bad over a week and ended up in hospital for 5 days - she left this early as she did not like getting IV antibiotics.

Many Participants missed hospital appointments which were not re-organised. Participant-3: *‘All those appointments about your health, you really don’t prioritise that.’*

Seven Participants reported not attending for HIV treatment and 14 reported losing out on the opportunity for treatment of their hepatitis C due to defaulting from appointments. Participant-46: ‘*Cause I was so busy getting drunk that I missed all me liver appointments and stuff like that. And now me liver is totally in bits.’*

Participants also frequently reported leaving the ED prior to either been see or prior to completion of assessment and management. Participant-3: *“Drunk one night and I must have hit my head against something but I....if I had...I’d to have 4 stitches or 4 stapes and I just left...I left the hospital and it closed up.”*

### Low usage of Primary Care Services. (22 sources/50 references)

Most people’s said they did not attend a GP as they had no medical-card. Participant-46: ‘*Cause some people’s would be too busy either getting stoned or drugged.....to actually send off these forms.’* However, even the possession of a medical-card (entitling one to free healthcare) did not guarantee the patient would attend the GP. Participant-8: *‘No one to make an appointment. It’s laziness. Just laziness. You know laziness and a drug addict.’*

### High (often described as inappropriate) usage of Emergency Departments (ED).

Again, as described in the literature, participants described situations where they used the ED in a manner that would be described by Health Planners as inappropriate. Firstly, they described using it for complaints that planners describe as being more suitable for primary care services (9 sources/14 references). Participant-47 had not been able to access health care except for the ED … as he had no medical-card and did not know how to get one*.* Secondly, they described using the ED purely for shelter and not for medical attention. (5 sources/13 references) Participant-18: “*I slept there for three months (laughs) … (laughs) … ..When I went in to the toilet I’d lock the cubical, put me sleeping bag out and went to sleep … And why Casualty. What … ..It’s Safe … and it’s warm, and it’s in out of the cold”*.

### Poor compliance with medication (10 sources/30 references).

A number of Participants reported not complying with (what would seem to be essential) medication. Participant-48 was supposed to be on aspirin as he had a history of having a stroke but had not collected the medicine in a few months.’ A few reported not taking their triple therapy for HIV. Participant-34: “*No, so I was thinking what’s the point?”*

### Avoidance of Psychiatric Services. (10 sources/ 17 references)

A number of participants who had mental health diagnoses reported avoiding attending their psychiatrist or avoiding taking their psychiatric medication. Participant-49 had an eating disorder, OCD and suffered from panic attacks. She did not want to see a psychiatrist as they had admitted her against her will on several occasions and she did not trust them. She had refused several attempts by her keyworker to link her with local GP’s, mental health services and public health nurses.

**Fig. 1:**
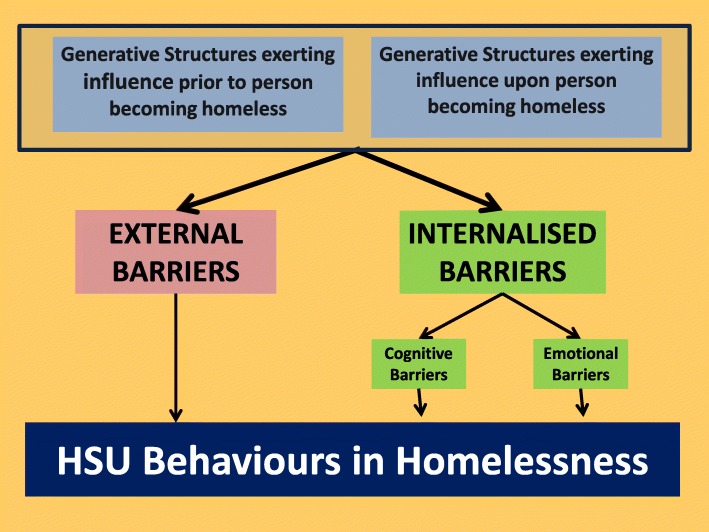
Critical realist model of HSU behaviours in homelessness

This study secondly, identified a number of mediators that influenced the behaviour of homeless people so producing the behaviour patterns identified above. These mediators are identified as barriers. External barriers are those that exist independently of the homeless people affected. Internalised barriers are barriers that operate through cognitions and emotions that affect homeless people’s health service usage behaviour but which originate from external social processes that affect individuals’ psyche causing them to use health services differently (Fig. 1).

The full range of generative structures, external and internal barriers are outlined in Table 3. In the following sections only evidence for findings rarely referred to or new to the literature is presented.

**Table 3:** Description of participants engaged through ethnographic fieldwork and focus groups

Participant ID	Gender	Description (note ages are estimates which can be unreliable in homeless population)	Where met
3	F	30-40 yo. hostel dwelling Intravenous Drug User (IVDU)	Focus Group
7	F	30-40yo. Hostel dweling underweight.	Food Hall
8	M	30-40yo IVDU rough sleeper	Street Outreach
9	M	20-30yo IVDU rough sleeper with schizophrenia.	Drop In Centre
11	M	30-40yo IVDU rough sleeper	Street Outreach
12	F	20-30yo Hostel dweling. IVDU	Food Hall
14	F	40-50 yo. Hostel dweling. Non Irish female socially isolated	Food Hall
15	M	40-50yo. hostel dwelling Drug User	Focus Group
16	F	30-40yo ex IVDU. History (hx) of HIV	Drop In Centre
17	F	40-50yo ex IVDU. Hx of HIV	Drop In Centre
20	F	20-30yo Junior doctor in hospital	Focus Group
22	F	20-30yo Junior doctor in hospital	Focus Group
24	F	20-30yo Junior doctor in hospital	Focus Group
25	M	40-50yo Hostel dweling with history of mental illness and alcoholism	Drop In Centre
29	F	20-30 yo. IVDU Rough Sleeper. Hx of Endocarditis and malnourished.	Street Outreach
31	M	40-50 yo. Hostel dwelling, ex- IVDU	Focus Group
32	M	40-50 yo. Homeless street drinker and rough sleeper. Unwell.	Street Outreach
35	M	40-50 yo. Hostel dweling IVDU. Malnourished and unwell.	Drop In Centre.
36	M	50-60 yo. Hostel dweling IVDU. Lot of medical issues.	Focus Group
37	M	30-40 yo. Hostel dweling IVDU.	Focus Group
38	M	40-50 yo. Hostel dweling ex IVDU.	Focus Group
44	M	30-40 yo. Hostel dweling IVDU.	Drop In Centre
47	M	50-60 yo. Rough sleeper, street drinker.	Street Outreach
48	M	60-70 yo. Hostel dweling. Street drinker.	Food Hall.
49	F	40-50 yo. Hostel dweling. Hx of severe anorexia	Street Outreach.
51	F	20-30 yo. Hostel dweling. Ex IVDU.	Focus Group
52	M	60-70 yo. Rough Sleeper.	Street Outreach.
55	F	20-30 yo. Rough Sleeper. IVDU. Severe arthritic condition.	Street Outreach.
56	F	20-30 yo. Rough Sleeper. From outside Dublin. Socially Isolated.	Street Outreach.
57	M	50-60 yo. Rough Sleeping. Hx of schizophrenia.	Street Outreach.
58	M	30-40 yo. Hostel dwelling. IVDU.	Street Outreach.
59	M	20-30 yo. Rough Sleeper. IVDU. From outside Dublin.	Street Outreach.
80	M	30-40 yo Hostel dwelling IVDU.	Drop In Centre.
81	F	30-40 yo. Hostel dwelling IVDU.	Drop In Centre.
82	M	40-50 yo. Hostel dwelling IVDU.	Drop In Centre.
83	M	20-30 yo. Hostel dwelling IVDU.	Drop In Centre.
84	F	20-30 yo. Hostel dwelling IVDU.	Drop In Centre.
85	M	30-40 yo. Hostel dwelling IVDU.	Drop In Centre.
86	M	20-30 yo. Hostel dwelling IVDU. Malnourished. Unwell.	Drop In Centre.
87	M	20-30 yo. Hostel dwelling IVDU.	Drop In Centre.
88	F	40-50 yo. Ex hostel dwelling – now housed. Ex IVDU.	Drop In Centre.
89	F	40-50 yo Hostel dwelling. Ex IVDU.	Drop In Centre.
90	M	20-30 yo. Hostel dwelling IVDU. Came from outside Dublin.	Street Outreach.
91	M	20-30 yo. Rough Sleeping. IVDU.	Street Outreach.
92	M	20-30 yo. Rough Sleeping. IVDU. Came from outside Dublin.	Street Outreach.
93	M	20-30 yo. Rough Sleeping. IVDU. Came from outside Dublin.	Street Outreach.
94	M	30-40 yo. Rough Sleeping. Street Drinker.	Street Outreach.
95	M	20-30 yo. Hostel Dwelling. IVDU.	Street Outreach.
96	M	20-30 yo. Rough Sleeping. IVDU. Deaf.	Street Outreach.
97	M	20-30 yo. Hostel dwelling.	Street Outreach.
98	M	20-30 yo. Hostel dwelling.	Street Outreach.
99	M	40-50 yo. Hostel dwelling.	Street Outreach.
100	M	30-40 yo. Hostel dwelling. IVDU.	Street Outreach.
101	F	30-40 yo. Hostel dwelling. IVDU	Street Outreach.
102	M	40-50 yo. Hostel dwelling. Moldovan.	Street Outreach.
103	M	20-30 yp. Rough Sleeper. IVDU. Domestic violence.	Street Outreach.
104	M	30-40 yo. Rough Sleeping. History of repeated violent behaviour.	Street Outreach.
105	M	30-40 yo. Hostel dwelling.	Street Outreach.
106	M	40-50 yo. Rough Sleeping. Street Drinker. Polish.	Street Outreach.
107	F	20-30 yo. Rough Sleeping. IVDU. Came from outside Dublin.	Street Outreach.
1108	M	40-50 yo. Rough Sleeping. Eastern European.	Street Outreach.
109	M	20-30 yo. Hostel dwelling. Eastern European.	Street Outreach.
110	F	40-50 yo. Couch surfing. Ex IVDU.	Food Hall.
111	F	20-30 yo. Couch Surfing. Ex IVDU.	Food Hall.
112	F	40-50 yo. Hostel dwelling American.	Food Hall.
113	M	40-50 yo. Hostel dwelling. IVDU.	Food Hall.
114	F	30-40 yo. Hostel dwelling. IVDU.	Food Hall.
115	M	30-40 yo. Hostel dwelling. Ex IVDU.	Food Hall.
116	F	40-50 yo. Rough sleeping. IVDU.	Food Hall.
117	M	40-50 yo. Rough sleeping. Street Drinker.	Food Hall.
118	M	20-30 yo. Hostel dwelling .Recent rough sleeper. Ex IVDU.	Food Hall.
119	M	30-50 yo. Hostel dwelling.	Food Hall.
120	M	20-30 yo. Hostel dwelling.	Food Hall.
121	M	20-30 yo. Hostel dwelling. IVDU.	Food Hall.
122	M	30-40 yo. Hostel dwelling. Ex IVDU.	Food Hall.
123	M	20-30 yo. Disliked doctors.	Food Hall.
124	F	20-30 yo. Hostel dwelling IVDU.	Food Hall.
125	M	60-70 yo. Ex homeless. Ex drinker.	Food Hall.
126	M	50-60 yo. Hostel dwelling. Mental Illness.	Food Hall.
127	M	40-50 yo. Ex Homeless. Ex IVDU.	Focus Group.
128	M	30-40 yo. Hostel dwelling Ex IVDU.	Focus Group.
129	F	20-30 yo. Hostel dwelling. Ex IVDU.	Focus Group.
130	M	40-50 yo Hostel dwelling Ex IVDU.	Focus Group.
131	M	30-40 yo. Hostel dwelling. IVDU.	Focus Group.
132	F	40-50 yo. Hostel dwelling. IVDU.	Focus Group.
133	M	40-50 yo. Hostel dwelling. Ex IVDU.	Focus Group.
134	M	30-40 yo. Junior hospital doctor.	Focus Group.
135	F	20-30 yo. Junior hospital doctor.	Focus Group.
136	F	30-40 yo. Junior hospital doctor.	Focus Group.
137	M	30-30 yo. Junior hospital doctor.	Focus Group.
138	F	20-30 yo. Junior hospital doctor.	Focus Group.
139	F	20-30 yo. Junior hospital doctor.	Focus Group.
140	F	20-30 yo. Junior hospital doctor.	Focus Group.
141	M	30-40 yo. Junior hospital doctor.	Focus Group.
142	F	20-30 yo. Junior hospital doctor.	Focus Group.

#### External barriers

There are a number of methods of categorising external barriers. This research adopted a categorization between physical, administrative, communicative, attitudinal and structural barriers (see Table 4).

**Table 4:** Generative structures, external and internalised mediators

Generative Structures. Structures Exerting Influence Prior to becoming homeless ● Poverty ○ Dysfunctional Familial Background *(23 sources/71 references).* ○ Substance Misuse *(8 sources/10 references).* ○ Negative Experiences of Authority *(35 sources/70 references).* ○ Illiteracy *(4 sources/4 references).* ● Mental Illness *(14 sources/22 references).* Structures Exerting Influence upon becoming homeless ● Insecurity of Accommodation. *(10 sources/19 references).* ● Need to Prioritise Survival over Health *(35 Sources/99 references).* ● Chaotic Nature of Homelessness *(26 sources/56 references).* ● Ubiquity of Early Death in Homelessness *(17 sources/39 references).* ● Ubiquity of Violence in Homelessness *(26 sources/78 references).* ● Substance Misuse *(42 sources/107 references).* ● Negative Experience of Authority *(35 sources/70 references).*	
External Barriers ● Physical Barriers ○ Distance *(6 sources/6 references)* ● Administrative Barriers ○ Complexity of application processes for medical care. *(12 sources/15 references)* ○ Appointment Systems *(16 sources/26 references)* ○ Queues *(18 sources/ 37 references)* ○ Policies for Management of addiction when queuing *(20 sources/48 references)* ○ Authoritative Rules of Service *(16 sources/39 references)* ● Communicative Barriers ○ Absence of Information *(10 sources/19 references)* ○ Conversations of Exclusion. *(36 sources/172 references)* ● Attitudinal Barriers ○ Stigma & Discrimination *(35 sources/222 references)*	
Internalised Barriers Cognitive Barriers ● Fatalistic Cognitions *(18 sources/47 references)* ● Denial Cognitions *(18 sources/45 references)* ● Presumption of Poor Treatment due to personal past experience or due to hearing of other people’s’s negative experiences *(15 sources/33 references)* ● Presumption of Discrimination Cognitions *(11 sources/33 references)* ● Self Blame Cognitions *(12 sources/37 references)* ● Competing Priorities Cognitions. *(35 sources/99 references)* Emotional Barriers ● Fear *(35 sources/96 references)* ● Hopelessness *(18 sources/36 references)* ● Embarrassment *(11 sources/30 references)* ● Low self-esteem *(8 sources/20 references)*	

#### Administrative Barriers

##### Rules of service

Breaking rules often resulted in barring that created barriers to accessing health services. Participant-9 (a homeless man with schizophrenia) had been barred from several services (including hostels and food halls) due to talking to himself loudly (a symptom of his mental illness). He had been living in a skip. In one hostel, staff said they knew he was mentally unwell but for the sake of other clients they felt they had no choice but to bar him.

A number of respondents reported being barred by their GP’s. Participant-18 “*No, I had a GP. He knocked me off … He just told me he didn’t want to be my GP anymore … ‘Cause I kept getting sick...I had him since I was four.”*

##### Policies for Management of Addiction in ED

A number of Participants left ED early due to not going into withdrawals from either drugs or alcohol as they were not provided with methadone or Librium medication to prevent such symptoms. Participant-23: *“The emergency.....you’d leave that to the last minute... because you’re left sitting there. It could be a day before they see you even, and most drug users have to get out...get money and ...drugs. I often had to (*leave the queue*) , I’d say most drug addicts do. When you come back you’re put at the end of the queue again.”*

Many Participants did not bother to go to the ED even with serious illness based on the presumption that they would not get methadone. Participant-7 had being very frightened as she had been told she possibly had TB due to changes on an x-ray done in the community. She was asked to come to hospital for a bronchoscope but refused due to fear of not getting methadone.

#### Communicative barriers

##### Conversations of exclusion

One particularly pernicious communicative barrier arose from particular repeated interactions between health professionals and homeless people where the homeless participant either was excluded or self-excluded themselves from the service. These were ascribed the term conversations of exclusion.

### The benzo conversation

In this conversation the homeless benzodiazepine taking person believes (not unreasonably) that doctors should give them benzodiazepines to prevent them going into withdrawals or to help them sleep. In fact a number of participants reported that homelessness was a factor in their insomnia. (5 sources/10 references) Participant-26: “*that is a lot of a strain...I needed them cos...it was the only thing that would get me to sleep,*?*”.*

The benzodiazepine conversation was described by Participant-16, a woman addicted to heroin and benzodiazepines who contracted both HIV and Hepatitis C. She described how once she would ask for benzodiazepines the doctor would angrily respond. This would cause her to become angry so she would ask them why not, and it would degenerate into an argument which would end with her leaving. Participant-17 said she had gone to doctors and *“once they hear you asking for benzos they get very angry.”* Participant-23 noted how once the doctor realizes you have a drug habit they presume you will be seeking drugs and will become frightened which then inhibits the conducting of a proper consultation.

The conversation can also be initiated by the doctor who predicts the patient will ask for benzodiazepines. Participant-12: *“Ah he’d say whatever you do don’t start asking me for Benzo’s”.*

Eventually the patient internalises this deterrent and develops a cognition that there is no point going to the health professional as they will simply presume you are looking for benzos and not treat you. Participant-15: *"I have problems in me family, and I’m anxious about that...I feel if I went in and told the doctor that, that he’d just turn around and say “Ah, he’s just looking for fuckin tablets...So I don’t bother … He sees me as a junkie.”.*

### The distrustful conversation

This conversation arises from a mistrust of homeless people’s behaviour in the consultation. As one doctor Participant-20 commented: *“So I felt very annoyed that someone that I had treated with respect was lying to get the prescription...You know you do your best, you treat someone with respect and then they turn around and they treat you like that. It will probably make me more suspicious, less trusting.”*

However, for homeless people as the health system would either not offer them what they felt they needed if they told the truth, telling a lie was essential to get their needs addressed. Participant-15: “*The thing about the drug addiction is...you do manipulate, you lie and you do coerce when you want that drug. You’ll say mass and you’ll promise the moon.”*

Other drug-using patients lied to avoid facing negative consequences. Participant-42*: “Well I don’t want to say it to me Methadone doctor.....about drink … Because he’d take me off me takeaways.”*

This presumption that trust was essential for a good relationship was not universal. In the research many key-workers never presumed participants would tell the truth and would not take offence if they were told a lie perceiving it as a normal and acceptable behaviour for homeless people.

### The blaming conversation

Some mainstream health providers believe that the health service is not for patients who ‘deliberately’ harm their own health in particular by using drugs. The blaming interaction is where the health professional blames the patient for causing their own health problems. Participant-46: *“They told me in the A&E that they couldn’t take me in because I was a drug addict and I made my own choices.”*

Even when the patient’s behaviour was not contributing to their medical problem they were being blamed. Participant-9 *“Because I was going in to the hospital and I was following up my treatment for me leg. It felt like “what are you still coming in for, you’re a Heroin Addict. You’re still injecting and all that”. I wasn’t injecting into me leg...I was injecting into my arms.”*

Participant-24 inadvertently owned up to blaming homeless drug users for not following up on their medical advice or appointments. *“I don’t get angry with people’s but I do get frustrated with them. From their point of view, they’re doing themselves a disservice.,, Yes, particularly where crossing the line is kind of a frequent thing and they come back again with the same problem...I’m just like Oh why didn’t you get it done? Something terrible could have happened to you.”*

### The assertiveness conversation

This conversation related to the fact that people’s from the mainstream housed population often had learnt the skills to assert themselves in a polite manner whereas homeless people often learnt ineffective methods of self assertion that lead to relationship breakdown with the service providers. Participant-25 was persuaded to go to ED for assessment of a head injury and I accompanied him. He walked up to the receptionist in an aggressive manner and immediately got into an argument where the receptionist looked frightened. He got frustrated and started to walk out. If the researcher had not acted as an intermediary he would have not received treatment. This demonstrated how aggressiveness is an ineffective assertiveness approach. Participant-50 described how he could get what he needed from services as he had been taught by his foster home how to be polite. He had learnt the discourse for traversing middle class health facilities.

#### Attitudinal deterrents and barriers

A number (9 sources) referred to the ‘disdain’ they experienced from health professionals. Participant-16: *“He just looked at me as if I was bleedin dirt like.”* Participant-14 was always buying new clothes to avoid looking homeless as she felt people’s discriminated against her when she ‘looked homeless’.

The drug-user stereotype (9 sources/25 references) seemed to be particularly pernicious. Participant-15: *“As soon as you give them your name, you know what way you’re going to be treated … they just don’t want to know once you’re a drug addict or an ex-drug addict.”*

Non-nationals outlined how they had experienced racist attitudes and behaviours from other homeless people though not from health professionals. This racism deterred them from attending many hostels or drop-in services. They used particular services that were perceived as being friendly to migrants e.g. the Capuchin food-hall.

#### Internalised barriers

##### Fatalistic cognitions

A number of Participants referred to their belief that they would not live very long so therefore what was the point in taking care of their health. Participant-41: *“What can you do?...If I’m going to die, I’m going to die.”*

Several respondents they believed they had a shortened life span. Participant-18: *“I don’t care about me life … I can see death, in me … I didn’t expect to live very long either.”*

Other people’s seemed not to care about their health and took risks that in the past had resulted in serious illness. Participant-29 had a history of a cardiac defect with subsequent bacterial endocarditis. Despite realising the risk of injecting heroin she had a fatalistic attitude: *“If it happens, it happens.”*

For Participant-62 it was not fatalism about his length of life about what he could achieve with what was left of his life. “*I feel...my life’s going to be shorter … I suppose I’m 36 years of age now. I’m not going to start raising a family now like. It’s a bit late for me to start now like.”*

##### Denial cognitions

A number of Participants simply denied the seriousness of their poor health. Participant-32 had been coughing up blood for a few months. He said he was too busy during the daytime to worry about seeing a doctor. As Participant-31 said: *“Everybody has a choice. I just wasn’t listening and was in denial with my health.”* Part of the denial cognition was that homeless participants seemed to have a different approach to analysing the risk/benefit ratio for taking health risks and attending health services (10 sources/17 references). Participant-35 described a swollen leg which sounded as if there was a high probability it could be a deep venous thrombosis (DVT). However, despite pointing this out, he decided not to go to for investigation as he thought it probably would not have been a clot and that it was a fair risk to take.

Several participants just did not want to hear bad news. Participant-14 recounted how she avoided going to see the doctor as it was coming up to Xmas and it was “*supposed to be a happy time with the kids.”*

A number of people’s with HIV described being in denial. Participant-11 missed her OPD HIV appointment. *"I will not let that fuckin disease beat me. I do think about it too and I do say “stop.. thinking about it.”* For her going to the HIV clinic *“makes it so real … Seeing all sick people’s around you.”*

One variant of denial was to defer managing serious health conditions till the future. Participant-7: *“obviously if I’d have gone to the doctor like, I would have probably been able to make a plan...thinking aw it’ll be get through tonight and then I’ll worry about tomorrow.”*

##### Presumption of poor treatment

Negative experience often resulted in participants refusing to attend the service in question as well as other similar services.

Participant-36 (who had a negative experience): *“Yeah I won’t go near that hospital.”*

Participant-38: *“Did you go to another hospital. Like if you were needing to go to the hospital? Did you go elsewhere?”*

Participant-36: *“No, I’m not going to any hospital.”*

##### Presumption of discrimination cognitions

Participant-23 said the presumption of discrimination was widespread amongst drug users. *“Most drug addicts think GP’s ..... if you go in with any illness as soon as they hear you’re on drugs they’re going to start looking down on them and they’re going to start talking down to them and treating them different … most people’s that’s on drugs … kind of keep away from doctors.”*

##### Self blame cognitions

A number of Participants believed that as their health conditions were related to their drug or alcohol usage that it was their own fault and they did not deserve treatment. Participant-39 decided not to attend the ED for a shortness of breath related to a chest infection as *“I just thought it was the drink, you know … I thought the doctor would just say “give up the drink” … Sometimes you feel like that too, only wasting their time, you know...It’s the way people’s look sometimes. Look on myself … It’s self inflicted", you know.”*

##### Competing priorities cogntitions

Many participants referred to having ‘survival’ needs that they prioritised over obtaining healthcare. Participant-3: *“You don’t prioritise yourself...It’s like, where are you going to get your next drugs, where are you going to get money. So that all comes first and the appointment doesn’t fit in, it’s just left aside.”* These priorities included obtaining shelter; drugs or alcohol; food; money from social welfare or begging; or consulting social welfare officers, childcare social workers, probation officers, key workers etc.

As well as cognitions a number of emotional states were identified that reduced the probability of using a health service. As with cognitions, these were aroused by external events or interactions and these were ascribed the term internalised emotional inhibitors.

#### Fear

Many Participants avoided services that were essential for their health due to fear of encountering aggression or violence. Methadone treatment centres were mentioned in particular. Participant-42: *“My partner like he wants off the Clinic...he was...jumped on...verbal confrontation and then bang...youngsters for some reason, the answer to everything is violence.”*

Emergency hostels were also perceived by many Participants to be dangerous. Participant-47 who was an elderly rough sleeping drinker was afraid of the drug users in these hostels and would not go in even when he had pneumonia. He chose to sleep rough in a Dublin suburb which as he said was “*All safe they are*. *quiet out there it is*” As many hostels had visiting doctors or nurses the choice to rough sleep affected access to primary-care.

Participants also referred to a generic fear of health professionals and the power they can wield. Participant-12: *“Yeah. And I’m very intimated by … big Doctors … I get very intimated around them. I start to get panicky and jumpy and can’t breathe … That power they have over you...they’ve the power to make you sick, they’ve the power to make you well and the power to intimidate you.”*

Participants defaulted from treatments either due to fear of investigations (e.g. Participant-42’s fear of liver biopsies) or fear of side effects of treatment (e.g. Participant-12’s fear of side effects of HIV treatment.)

#### Hopelessness

Hopelessness about what one could expect in future life also acted as a barrier for attending healthcare. Participant-18: *“I don’t care about me life.”*

Repeated failure instilled a sense of hopelessness. Participant-27: *“I started back drinking that’s when...I’s probably change into someone else, not necessarily a bad person like but just careless and not care about things or make appointments and all like...I’d always be starting fresh somewhere...I’m going to get my shit together and all and then I’d be back to square one in a a few weeks.”*

#### Embarrassment

Embarrassment due to poor personal hygiene was a cause for avoidance of attending health services. Participant-6 did not attend with his deep venous thrombosis due to embarrassment at his appearance. ***“****Yeah, yeah, you know what I mean because I was dishevelled … when you’re homeless and in that situation...I was sleeping the street for a week and you can’t* (go into hospital like that).”

#### Low self-esteem

Participant-15 did not go to hospital with the clot in his leg. “*I think a lot of addicts have an inferiority complex … I know for a fact that when you go into that hospital, you have all that burden on your back. … You do feel very small within yourself...Never mind the doctors that you feel lower and less of a life form than them. That you leave it that late.”*

Low self esteem also prevented Participants asserting themselves when they felt poorly treated. Participant-4: “*Well I don’t speak up for myself the way other people’s would.”*

#### Generative mechanisms for homeless people’s HSU

A number of generative mechanisms for the HSU of homeless people were identified. Some of these factors preceded Participants becoming homeless while the majority had their effect while the Participants were homeless.

#### Generative factors preceding homelessness

##### Poverty generated a number of the factors that influenced the HSU of Participants

The vast majority of the homeless people encountered originated from the deprived areas of Dublin. Poverty affected Participants’ HSU in several ways.

##### Familial dysfunction

Participants referred to dysfunctional familial backgrounds with stories of familial breakdown, domestic violence, parental drug and alcohol misuse, physical and sexual abuse. Partcipant-5 *“*(My dad died of) *the virus … when I was 7 though, my ma died when I was 18 months* (of an overdose) … .*then my stepfather took me and then they got … .divorced and then …*. *my step ma went on heroin so … .my stepfather … started abusing me.”*

Familial dysfunction often resulted in participants ending up in care. Participant-4: *“I’m in care since I’m six. Me mammy...wanted to go away with her partner and he abused me...and hit me as well. He still beats me Mam...She brought me to the Social Worker and she left me suitcase there and then she left a note at the counter.”*

##### Substance misuse

Many Participants had experienced substance misuse prior to becoming homeless either due to mis-using themselves or due to a family member mis-using substances. Participant-51 described how both of her parents were drug users and her mother had introduced her to drugs when she was a pre-teen. Her mother than died from HIV when she was a teenager and then she got pregnant when she was 17.

##### Fear/mistrust of authority

Many Participants had negative past experiences of social authorities. Many had experienced being in prison, usually on repeated occasions (14 sources/18 references) where their experience of authority figures was very negative. Social workers evoked much distrust amongst participants. There were frequent stories of children being put into childcare. (15 sources/ 27 references). Participant-19’s ex-girlfriend had committed suicide: *“She couldn’t take it cause I was locked up. The kids were taken away from her.”*

##### Illiteracy

Illiteracy affected some Participants’ ability to engage with health services. Participant-13 told of how he had disengaged from his Hepatology clinic. Due to illiteracy he never checked his post and missed his clinic appointment.

##### Severe mental health problems that cause homelessness also affect HSU

In this study a number of homeless people with mental health problems refused to engage with health services. Participant-52 slept in a doorway for over 3 years. He had a flowing white beard and wore typical tramp clothing with several coats and a tweed hat. He told everyone who approached him including me to fuck off. The only service he engaged with was to go for his dinner to a local food hall. A psychiatrist came to do an assessment but he refused to engage. He was admitted to a hospital eventually where he was diagnosed with schizophrenia 8 days before he died.

#### Generative factors taking effect while homeless

##### Lack of appropriate accommodation affected HSU

In the homeless sector where people’s spent their nights had a significant effect on their health service usage as well. As Participant-31 said: *“most of us just need a home to start us off.”*

Participant-1 had a chronic severe leg ulcer (covering half of both his lower legs) which if not dressed daily would deteriorate, become infected and incredibly smelly: “W*hen I wasn’t living anywhere permanent … I would miss but I’d be annoyed with myself and then I’d … .then when I would go to* (the nurse) *and I would be a really bad … the smell.”*

The concept of homelessness embraces a range of accommodation statuses ranging from those sleeping rough in the streets, to those in emergency hostels and those who were couch-surfing. Each of these scenarios had effects on the HSU of Participants.

Rough sleepers lived in the harshest circumstances (24 sources/55 references).

It was clear that in a rough sleeping environment the maintenance of health dropped down the priority list. Participant-57 who had a spinal deformity, poor mobility, malnutrition, double incontinence and a history schizophrenia had slept rough for 15 years in a park in a wealthy Georgian square in a Dublin suburb. He was sleeping in an igloo that some local teenagers had built in the park. He had not medical card and had not seen a doctor in several years. Participant-55 missed hospital appointments when rough sleeping and her rheumatoid arthritis had caused irreparable damage. Rough sleeping meant that Participants could not wash or clean themselves and like Participant-47 felt self conscious if queuing in waiting rooms.

The presumption that a hostel would be better than rough sleeping was often refuted by clients who chose not to stay in hostels (21 sources/34 references). Participant-6 said the hostels he was offered were always dangerous and poorly run: *“I’d rather … .to be honest … I know this is like the extreme …*. I*’d rather be found dead in the street than in a hostel to be honest with you.”* Participants referred to how hostels often failed to take account of their medical needs. Participant-56 was in severe pain from sciatica and had to use crutches. She broke down in tears when describing how she had gone to the ED and was told to have bed rest but she had to leave hostel at 9.30 to 16.00. She spent her days in a library during day in pain.

##### Ubiquity of premature death affected attitude to HSU

One of the determinants of fatalism amongst homeless people was the ubiquity of early death. Three people’s died during the course of the research. Each week there were stories circulating about new recent deaths. Participant-13 described how his best friend had just hung himself in prison. He added that 4 other people’s died that same week, one from snow blow, and 3 others had collapsed but no cause had yet been identified. His opinion was they had probably overdosed.

##### Substance misuse was endemic and affected HSU

The obtaining of drugs and/or alcohol was often prioritised over HSU (15 sources/33 references). Participant-59 commented: “*you don’t give a fuck about your health - all you want is the drugs.” Participant-51* added: *“I suppose when you’re so bad on drugs … You have no organisation in your life plus you’re very forgetful..So, one, you don’t really have a lot of respect for yourself and you wouldn’t have much organisational skills … To go to appointments and to be on time and stuff like that.”*

Many Participants (9 sources/15 references) used drugs or alcohol to treat the symptoms and so avoided HSU. Participant-1 took heroin to ease the discomfort caused by his leg ulcer:

##### Immediate survival was prioritised over HSU

Taking into account the high rates of premature death and level of violence, it is not surprising that participants said their main aim when homeless was to survive (12 sources/17 references). Participant-41: *“It’s holding on to the fuckin’ rope and not letting go, you know what I mean.”*

##### Threat of violence affected HSU

A number of Participants had observed violence. (7 sources/13 references) Participant-48 described how he witnessed a fellow rough sleeper die in a hostel due to an assault: *“They thought he was drunk and they said “either get out and walk it off or go to bed”...You’re not supposed to go asleep with a head injury... and he never woke up the next morning. He got a dig with a knuckle duster into the head.”*Many participants had experienced violence (15 sources/32 references). Participant-29 outlined how violence was *“to be expected”* when one is homeless. Repeatedly participants reported how the level of violence encountered outside addiction treatment centres created a deterrent to HSU at those centres (18 sources/28 references).

##### Chaotic nature of homelessness affected HSU

The chaotic nature of homelessness led to the development of many internalised barriers. Participant-7: *“Just drinking, doing drugs, just living in different hostels and then getting my own place and then not being able to keep it up and just going around in circles really like...when I left care like, I didn’t really know how to live like a normal person … Then I started drinking heavily and then not paying rent and then just one thing led to another and then back on the streets and … .back into the vicious circle like.”*

The negative effect of chaotic lifestyles on engagement with health services is particularly notable for homeless people. Participant-25: *“I suppose you feel – I know there are chaotic lifestyles and things like that. You don’t follow up with an appointment or a scan sometimes”.*

##### Negative experiences of social authority

When homeless, participants had experiences with authority that often mirrored and re-enforced earlier negative experiences of authority. A number had been barred from hostels or had very negative experiences with social welfare and social work systems. A number described bad experiences with authoritative medical professionals. Participant-44 described how his GP had picked up his prescription and said “*if I want I can tear this up*”. He said: “*he tore it in front of me*. *I said what sort of Doctor would do that*”. He described the doctor as: “*was acting like God*”.

## Discussion

This research was concerned with making sense of the seemingly chaotic nature of homeless people’s HSU. Homeless participants in this research experienced biographical events that distanced them from mainstream institutions and resulted in them existing in the chaos of homelessness. Their HSU became shaped by encountering a range of physical, administrative, communicative and attitudinal barriers and further internalising a series of cognitive and emotional barriers. The final result was discordance between mainstream’s health service utilization behavioural norms and the actual behaviours displayed by homeless people.

In light of the invidious mortality and morbidity statistics for homeless people, the seemingly inefficient and counter-productive engagement with health services seems ‘senseless’ to health professionals. The resulting frustration is exacerbated by the fact that these very behaviours place a significant burden on healthcare institutions and their staff. As Moore et al. (2007) noted *“health service providers have limited resources, flexibility and understanding to help the homeless.”* [29] This creates the risk that medical staff rather than continuously seeking to understand will place their own interpretation and judgement of the behaviours and produce the concept of the’ inappropriate attendee’.

This concept implies the existence of a converse social construction i.e. the ‘appropriate attendee’. Such an entity presumably knows how doctors believe ED should be utilized i.e. sparingly, only for serious conditions and after seeing a GP where possible. Homeless people rarely adhere to this construction. This can lead to ED staff judging them as ‘wasting’ their time and the department’s resources. Feldman et al. (2017) noted that there was *“abundance of literature about homelessness in the ED...more focused on ... the relationship of homelessness and frequent utilization of resources, excess cost of care for the homeless, and...relating to homeless people’s way of being can become negative.”* [30]

We could surmise that from homeless people’s perspective, the ideal health service would provide primary and secondary care, not require appointments, be open 24 h, allow patients to wander in and out of the waiting room and provide places to rest and sleep that are superior to a street alleyway or pavement outside a shop entrance i.e. the ED. Moore et al. noted that *“the utilisation of ED by homeless people is not about inappropriate use but about how homeless people manage their health issues and survive a chaotic life style.”* [29]

This gap in understanding between service providers and homeless people is particularly evident in Conversations of exclusion. This concept is unique to the literature and as such do contribute to untangling the complex causation for homeless people’s HSU. It is known that there is tension in the relationship between doctors and homeless patients when it comes to prescribing benzodiazepines. Doctors feel ambivalent, being caught between the opposing impulses to either prescribe so as to please the patient or shorten the consultation or not prescribe due to the addictive potential and desire not to been seen as over-prescriber amongst one’s peers [31–33]. On the other hand drug mis-users feel that they are the ‘experts’ on the effect of addiction on their personal lives [34]. It is further known that homeless people have to resort to deception or ‘trickery’ in order to survive. Scamming, ruses, criminal activity are all part of the survival strategy for homeless people. This distrust often leads to exclusion for homeless people from services [16]. Doctors have been warned to be wary of drug users manipulative behaviour and to adopt a distrusting stance in such relationships [35, 36]. Lastly, it is known that homeless people have difficulty asserting themselves [37] and an *‘expectation of rejection and anger can be quite near the surface...(that)...can spark off aggressive behaviour.*’ [38] Such challenging behaviours have been noted to disrupt their access to health services [39].

The literature on homelessness has concentrated on making sense of homeless people’s HSU by attributing its causation to external barriers of which many have been identified in this research including distance/lack of transport [40, 41]; complex administrative processes [9, 42, 43]; forms [44]; appointments [11, 41, 44, 45]; queues [11]; restrictive rules [38, 46]; lack of information of how to access services [37, 44]; illiteracy [47]; and attitudinal barriers [44, 48–50]. The barrier created by ineffective management of addiction withdrawals in ED is not referred to in the literature. This may because it is a phenomenon that is unique to Dublin.

Internalised barriers are a new concept to the literature that help extend our understanding of why homeless people seem so apathetic about attending health services. Supportive evidence exists for such barriers:

- Several authors have noted homeless people displaying fatalistic attitudes [51].
- The witnessing of many young deaths can lead to the development of a sense of living in the moment, taking 1 day at a time [52].
- Both poverty and homelessness are associated with denial of having serious physical and mental illness [53, 54]. This is known to inhibit HSU [55].
- Homeless people have been found to be distrustful of the treatment they will receive due to poor previous experiences [47, 48, 54].
- Many homeless people expect to be stigmatised often due to previous discriminatory experiences and so avoid engaging with heath services [51, 54, 56].
- Homeless people have a tendency to self-blame including taking blame for being homeless [57]. Social stigma has been identified as a particularly powerful cause of this internalised self-blame [58, 59].
- Homeless people live their lives both on the street and in hostels in persistent fear of assault; stigmatization; having their possessions stolen and of their children being put in care or becoming involved in drugs or crime [20, 36, 60, 61].
- Fear of authority figures including health professionals and social workers as well as fear of a serious diagnosis are recognised deterrents for homeless people’s usage of services [37, 61, 62].
- Fear of aggression is recognised as a deterrent for use of services [63].
- It is recognised that homeless people often feel hopeless; in the control of external forces; and with no optimism as to their future prospects [64].
- Poor personal hygiene causing embarrassment has been recognised in the literature as a cause for avoidance of health services [33].
- Low self-esteem has been identified as one of the major causes for homeless people’s low usage of health services [42]. Stigma has been identified as one of the main causes of low self-esteem in homeless people [37].
- Poorly drawn up rules of service can exacerbate this potential for anger and confrontation [38].

The generative structures proposed by this research have support in the literature. The origins of an explanation for homeless people’s HSU often lay in their background in poverty [65, 66]. Poverty causes huge stress on families thus increasing the likelihood of those families becoming dysfunctional [67]. Many homeless people originate from dysfunctional familial backgrounds [67, 68]. Dysfunctional familial backgrounds have been recognised as a cause of poor engagement with health services [67, 69]. Substance misuse is endemic in areas of deprivation [70]. As in the literature, many participants were using drugs prior to becoming homeless [71]. Substance mis-users are less likely to engage with health services due to the chaos created in their lives by the addiction [70]. Sadly, health services are also unlikely to seek engagement with homeless substance mis-users with many even refusing to accept them as patients [42]. In the literature on poverty and homelessness, fear or distrust of authority figures is a well recognised reason for homeless people avoiding consulting health professionals [11, 49]. Negative experiences of the justice system, housing authorities and of having children put in care have been noted to inhibit engagement with health services [72]. Lastly, poverty and homelessness are associated with high rates illiteracy [73]. Illiteracy affects HSU [74].

Mental illness both causes homelessness and acts as a deterrent for engagement with health services [41, 75].

It is known that for homeless people, death is ubiquitous and could always be just round the corner [51]. Substance misuse is pervasive among the homeless population being both a cause and a consequence of homelessness [7]. Violence is likewise a daily threat that homeless people face [48]. It is known that substance mis-users are less likely to engage with health services often due to the chaos created in their lives by the addiction [76]. This fight to survive affected HSU as homeless people prioritised more immediate needs that they saw as more important for day to day survival than health. This is referred to in the literature as competing priorities [44, 48, 77]. The negative effect of chaotic lifestyles on engagement with health services is particularly notable for homeless people [78].

### Model of HSU for homeless people

**Fig. 2:**
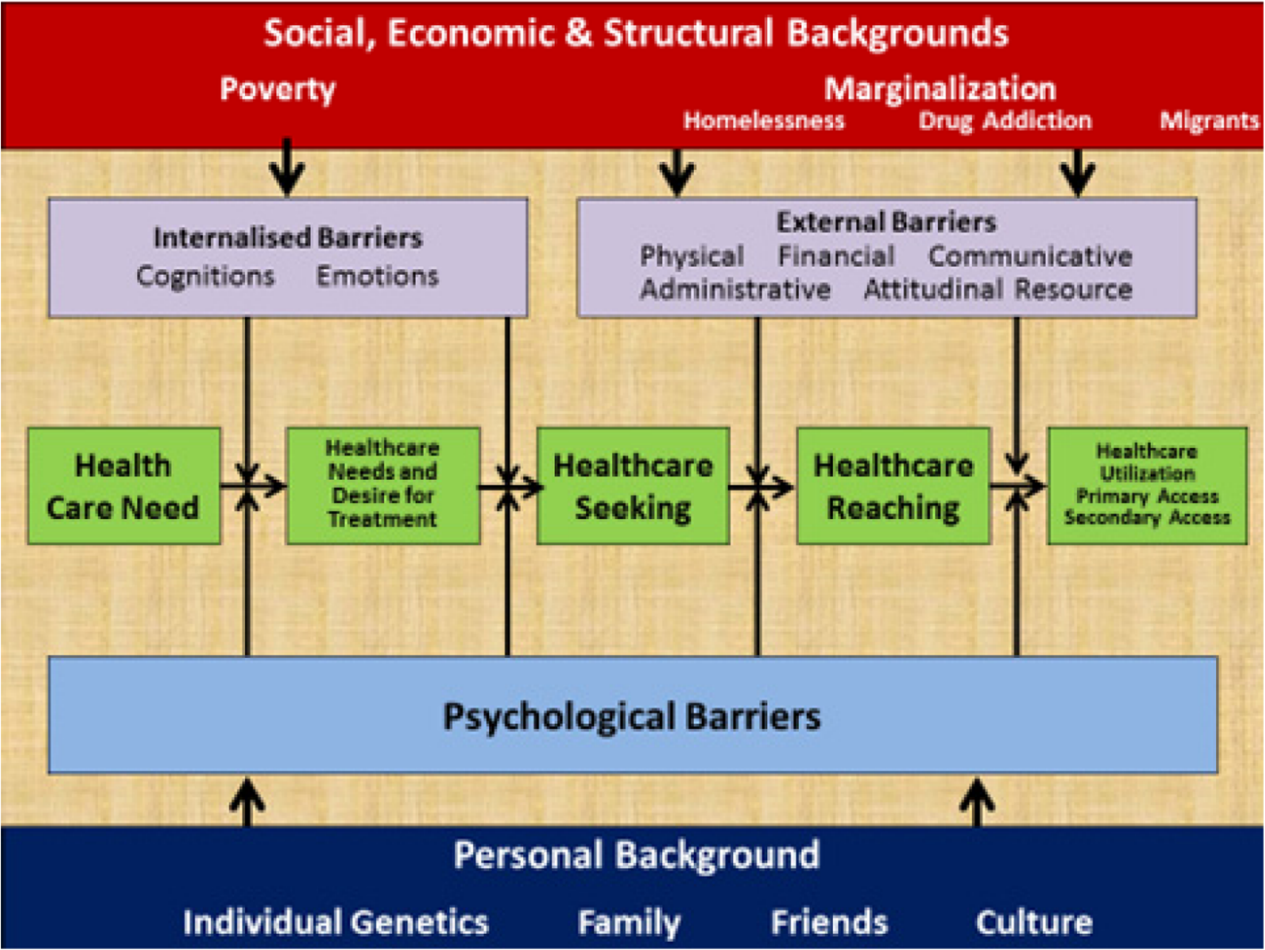
Critical realist explanatory model for why homeless people use health services differently to domiciled people’s

A critical realist model was devised based on the findings of this research that offers an explanation for why homeless people have a differing Health service Usage behavior from the domiciled population (Fig. 2)

This model borrows from that of Levesque et al. (2013) in that it places the journey from symptom recognition to end service utilization [79]. It seeks to identify the barriers that act at different points of this journey that result in a particular health decision making and health service usage behavioural pattern. On the bottom are factors that affect all people’s that contribute to personal psychological barriers e.g. a familial fear of illness may act as a psychological barrier for a homeless or a housed person. Mental illness is a particular issue that can contribute to individual psychological barriers that can affect both homeless and housed patients but due to the high level of mental illness in homeless populations has a particular invidious effect for homeless patients.

On the other side the internalised and external barriers take effect at different points on the journey. At the top are the proposed generative mechanisms that create the conditions for the external and internalised barriers to arise and operate.

A number of models seeking to make sense of homeless persons’ HSU are described in the literature. The Gelberg-Anderson Explanatory Model for Homeless people’s HSU proposed a model with predisposing; enabling and need factors as well as recognising the effect of actual health behaviour on future behaviour. They created domains applicable to all patients and domains specific to vulnerable populations (including homeless people) [11]. (see Table 5).

**Table 5:** Gelberg Anderson Behavioural Model for Vulnerable Populations [10]

Predisposing Factors	Enabling Factors	Need	Health Behaviour	Outcomes
Traditional Domain				
*Demographics* Age Gender Marital Status. *Health Beliefs* Values Concerning health and illness Altitudes towards health services. Knowledge about disease *Social Structure* Ethnicity Education Employment Social Networks Occupation Family Size Religion	*Personal / Family Resources* Regular Source of Care Entitlement to free primary-care Income Social Support Perceived Barriers to Care *Community Resources* Residence Region Health Services Resources	*Perceived health* General Population health conditions *Evaluated Health* General Population health conditions	*Personal Health Practices* Diet Exercise Self Care Tobacco Use Adherence to care *Use of Health Services* Ambulatory Care Inpatient Care Alternative Healthcare Long-term healthcare	*Traditional & Vulnerable Domains* *Health Status* Perceived Health Evaluated Health *Satisfaction with Care* General Satisfaction Technical quality Interpersonal aspects Coordination Communication Financial Aspects Time spent with clinical Access / Availability Convenience Continuity Comprehensiveness Administrative Hassle
Vulnerable Domain				
*Social Structure* Country of birth Acculturation / Immigration / Literacy Sexual orientation *Childhood characteristics* Residential History (e.g. foster care, orphanages) / Homelessness Living Conditions Mobility (stability of home) Length of time in the community Criminal behaviour / Prison history History of abuse or neglect Mental Health Psychological resources (self esteem, social skills etc) Substance abuse	*Personal / Family Resources* Competing Needs Hunger Social Benefits Self Help Skills Ability to negotiate system Key worker Transport Telephone Information Sources *Community Resources* Crime rates Social Services resources	*Perceived health* Vulnerable Population health conditions *Evaluated Health* Vulnerable Population health conditions (e.g. TB, STD’s, and HIV, Hepatitis etc and substance abuse and mental health problems).	*Personal Health Practices* Food sources Hygiene Unsafe sexual practices	

Penchansky & Thomas (1981) sought to integrate both demand and supply sides by positing that health service usage depends on the “fit” between individuals (clients) and the health care system." This fit can be measured in terms of service availability (has the provider the necessary resources to meet the needs of the client); accessibility (i.e. geographic proximity); affordability; accommodation (are the services organized to meet address constraints faced by client); and acceptability (how comfortable the client feels with the provider individuals and organization) [80, 81].

The Institute of Medicine created a framework for monitoring HSU behaviours in the early 1990’s. This model had a more explicit focus on monitoring and comparing access for differing groups so as to identify where inequitable access existed and what potential financial, structural or personal barriers would explain such a discrepancy [82].

Levesque et al. (2013) integrated several previous models, including a pathways models constructed around a patients journey from identifying a health care need to the consequences of accessing a service; Penchansky & Thomas’s concepts of affordability, acceptability, availability and accommodation; while adding in two further categories of approachability (the ease at which a patient can identify a suitable service) and appropriateness (the suitability of the service provision for meeting the patient’s needs). Lastly, they matched categories on the supply side with five abilities on the demand side that derive from the determinants models. Levesque et al. were attempting to both identify an operational measure of access and also to guide researchers wishing to measure access and policy-makers seeking to improve access [79].

The model that is proposed in this research differs from previous ones. Firstly, it was developed from research whereas previous models were developed a priori and then tested through research. Secondly, it does not attempt to explain or predict homeless people’s HSU but rather to explain why their HSU differs from that of the general population. The effectiveness of previous models ability to predict HSU has been questioned due to lack of sufficient evidence [83, 84]. Thirdly the model includes the concept of internalised barriers and conversations of exclusion not present in other models. Lastly, being a critical realist model it describes a number of generative mechanisms.

It is suggested that this model is of particular use for service providers who are seeking to improve accessibility for their services. It offers them a framework to firstly audit potential external physical, administrative, communicative and attitudinal barriers. It further makes them aware of the need to explore not only current service users perspectives of the service but also ‘potential’ users who may have internalised barriers created from past experiences of that particular, or similar services. Such exploration would include needs analysis or surveys amongst potential users of a service.

The model also seeks to make policy makers aware that the when engineering health services they need to model the services on the health service usage behaviour of all potential users and not simply the majority. The model further creates awareness of how those behaviours originate not from ‘recalcitrant’ homeless individuals but from the social processes that create the conditions of poverty and homelessness itself. Crisis (2002) noted that the systems of health service provision were one of the main contributors to poor access for homeless people [85].

This research suggests that the creation of a single accessible health system, while conceptually desirable, may be pragmatically unachievable as it would require a total redesign of our present system addressing the wide range of external barriers and addressing deeply seated prejudicial attitudes as well as overcoming a range of internalized barriers. As Moore et al. comment *“health services need to understand the difficulties faced by people’s who are homeless and that standard health services do not meet their needs.”* [29] While the services should seek to develop full accessibility, adaptations that make our present system accessible such as ‘specialised’ services may need to recognized as an adaptation of the mainstream services that enables access for homeless people. Jones & Pleace noted that *“services have obviously been developed or modified to counteract the known attitudinal and organizational problems that were blocking access to the NHS for homeless people.”* [45] These services are undoubtedly favored by homeless people [86, 87]. There is also significant evidence that such services improve access to health care for homeless people both internationally and in Ireland [8, 48, 87–89]. When offered as a part of a multi-faceted response ‘specialized’ health services can help offer a route out of homelessness [90].

## Conclusions

The HSU of homeless people in Dublin resembled that of HSU of homeless people internationally. The HSU of the participants in this Dublin based research differed from that of the domiciled population dueto a range of external and internalized barriers. The origin of these barriers was traced to generative mechanisms that arose from both poverty (where most participants originated from) or from homelessness itself. These findings were integrated into an explanatory model for why homeless people’s HSU differs from that of the general population.

### Limitations of the research

There are a number of limitations to this study:

- Firstly, this research was conducted in Dublin and care must be taken in transferring findings to other populations.
- Secondly, the researcher, was an insider in the field prior to commencing the research as a doctor who attended many of the homeless people encountered when in the research role. This may have limited their interactions and opinions Participants offered me.
- Thirdly, the researcher founded and worked in a number of specialised services for homeless people. This could bias his opinions of specialised services which were viewed in a favourable light within the research.
- Lastly, the researcher was sole coder and interpreter of the data collected during the research.

## Data Availability

The datasets used and/or analysed during the current study are available from the corresponding author on reasonable request.

## Abbreviations

ED: Emergency Department
GP: General Practitioner
HIV: Human Immunodeficiency Virus
HSU: Health service Utilization
OPD: Outpatient Department
